# Interventions to Reduce Repeat Presentations to Hospital Emergency Departments for Mental Health Concerns: A Scoping Review of the Literature

**DOI:** 10.3390/healthcare11081161

**Published:** 2023-04-18

**Authors:** Wanying Mao, Reham Shalaby, Vincent Israel Opoku Agyapong

**Affiliations:** 1 Department of Psychiatry, University of Alberta, Edmonton, AB T6G 1C9, Canada; rshalaby@ualberta.ca (R.S.); vn602367@dal.ca (V.I.O.A.); 2 Department of Psychiatry, Faculty of Medicine, Dalhousie University, 5909 Veterans, Memorial Lane, 8th Floor Abbie J. Lane Memorial Building, QEII Health Sciences Centre, Halifax, NS B3H 2E2, Canada

**Keywords:** readmission, emergency department, psychiatry, mental health, intervention, patient

## Abstract

Background: The number of readmissions to the emergency department (ED) for mental health services each year is significant, which increases healthcare costs and negatively affects the morale and quality of life of patients and their families. Object: This scoping review aimed to establish a better understanding of interventions that have been implemented to reduce psychiatric patient readmission and ED use within the ED, to identify areas for improvement, and therefore to assist in the development of more effective actions in the future. Method: The scoping review was conducted on several bibliographic databases to identify relevant studies. Two researchers independently screened and reviewed titles, abstracts, and full-text articles that met the inclusion criteria. Using Covidence software, 26 out of 6951 studies were eligible for inclusion in this scoping review based on the PRISMA checklist. Data were extracted, collated, summarized, presented, and discussed. Result: This review identified 26 studies which examined interventions aimed to reduce ED visits, such as the High Alert Program (HAP), the Patient-Centered Medical Home (PCMH), the Primary Behavioral Health Care Integration (PBHCI), and the Collaborative Care (CC) Program, etc. Twenty-three of the studies were conducted in North America, while the rest were conducted in Europe and Australia. A total of 16 studies examined interventions directed to any mental health conditions, while the rest addressed specific health conditions, such as substance use disorders, schizophrenia, anxiety, depression. Interventions involved comprehensive and multidisciplinary services, incorporation of evidence-based behavioral and pharmacological strategies, and emphasized the case management that was found to be effective. Additionally, there was a marked consideration for diverse mental health groups, such as those with substance use disorder and of young age. Most interventions showed a positive effect on reducing psychiatric ED visits. Conclusion: Various initiatives have been implemented worldwide to reduce the number of emergency department visits and the associated burden on healthcare systems. This review highlights the greater need for developing more accessible interventions, as well as setting up a comprehensive community health care system aiming to reduce frequent ED presentations.

## 1. Introduction

Often, mental illness affects an individual’s perceptions, emotions, and behaviors, as well as their performance at work, in social settings, or in other areas of everyday life [1]. According to World Health Organization estimates, nearly a billion people worldwide suffer from mental illnesses, which makes mental illness the most common cause of injury and disability [2]. In 2012, the Mental Health Commission of Canada reported that currently there are more than 6.7 million Canadians living with a mental disorder or illness, with approximately 20% experiencing mental illness at some point in their lifetime [3]. Aside from being common, mental health conditions are also expensive. According to data indicated in *Public Expenditures for Mental Health Services in Canadian Provinces* published in 2013, the Canadian government spends CAD 7 billion annually on these services [4], with mental health related Emergency Departments (ED) services costing about CAD 2.7 billion annually, making them one of the five most expensive patient groups due to the large volume of visits [5].

EDs play a crucial role in the delivery of health care. Globally, EDs have seen ever-increasing volumes of patients seeking psychiatric treatment over the past decade across varied populations and contexts [6]. For example, adult ED visits for mental health disorders increased by over 30% in the United States between 2006 and 2015. The majority of these visits were related to alcohol use disorders, followed by mood disorders and anxiety disorders [6]. A similar trend was also observed between 1992 and 2001 [6,7]. In Australia, the number of ED visits concerning mental health issues increased between 2004/2005 and 2016/2017, largely due to psychoactive substance abuse which involves drugs or other substances that affect mood, awareness, thoughts, feelings, or behavior such as alcohol, caffeine, nicotine, and certain pain medicines, etc., followed by anxiety and mood disorders [8]. Between 1988 and 2014, Taiwan saw a rise in visits to ED associated with depression disorders, suicide attempts, as well as trauma and stressor-related disorders, which are emotional and behavioral problems that may be caused by childhood traumatic and stressful experiences [9]. On the basis of available data, research in Canada is consistent with trends in the USA, with anxiety disorders and substance abuse disorders showing the most upward trends [6].

Recurring visits to the ED for mental health issues are common [10,11,12]. One in five patients presenting to the ED for a mental health concern have a repeat visit within six months [13]. Half of all mental health related hospital admissions and 60% of hospital discharges are associated with repeated ED visits for the same or similar complaints [14,15]. These so-called frequent ED visitors account for a small number of ED patients (4.5 to 8%) but comprise 21 to 28% of all ED visits [16]. What is meant by the term “frequent visitors” has a wide range of definitions. According to the majority of research studies, people who are frequent users of the ED have had four or more visits in the previous twelve months, whereas some groups define them as people who visit the ED twice in a year. Others define them as those who visit 12 times a year [17,18,19]. This scoping review included papers that defined and discussed “frequent visitors” in a wide range of ways.

Research has indicated that frequent ED users who suffer from recurrent mental health problems have a 25–30% likelihood of experiencing acute psychiatric illness [14,15,16,17,18]. Recurrent ED users with mental health issues were most likely to be diagnosed with personality disorders, depression, and anxiety [16,17]. It has been reported in the literature that frequent users of EDs have a disproportionately low socioeconomic status, are males, members of minority groups, chronically ill, alcoholics, frequently presenting with psychiatric or substance abuse problems, and have a lack of social support. Chronic substance abuse, being single, being homeless, and not having social health insurance were reported predictors of recurrent ED visits due to acute mental health problems [16,20,21]. Repetitive ED visits are associated with overcrowding, unnecessary delays in care, dissatisfaction, and avoidable patient harm [22]. Because the ED is also an expensive place for medical care, repeat visits can contribute to high healthcare expenditures. ED care costs are estimated to be between two and five times higher than that of primary care. In these situations, patients and their families suffer not only significant physical and psychological consequences but also financial consequences [22]. The health system is also negatively impacted by a lack of infrastructure, human resources, and financial resources caused by repetitive ED visits [23]. One report indicates that repetitive ED visits are one of the significant factors causing ED crowding, which results in increased violence towards staff, a high turnover rate of clinicians and nurses, decreased provider productivity, high ED physician burnout, and increased staff distraction, which is a key contributor to high ED physician burnout, approaching 75% [23]. In addition, in a systematic and narrative review of studies from different countries, several studies indicated substantial savings in hospital costs associated with a decrease in ED visits [24]. Worldwide, avoidable ED readmissions are becoming increasingly prevalent.

From the literature several initiatives have been proposed to close the gap of frequent ED use by patients with mental health conditions; however the rates of ED admissions are still high posing a threat to the capacity of healthcare systems globally. The effectiveness and the implementation capacity of the interventions used represent a prime interest and a critical area that need to be closely addressed, aiming to close this growing gap and achieve overall better health and non-health outcomes. Therefore, this study aimed to examine interventions that have been implemented to curb repeat ED visits by patients for mental health concerns.

The specific goals were as follows:

(1) To identify the scope, nature, distribution, and different types of the interventions that have been used to curb repeat ED visits by patients.
(2) To investigate these interventions based on the mental health diagnosis received.
(3) To examine the effectiveness of these interventions in reducing the total number of ED visits and/or the length of stay (LOS) in the ED.

## 2. Methodology

### 2.1. Study Design and Search Strategy

This scoping review followed a systematic search strategy, which was developed and applied to electronically conduct a data search in several major scientific databases. These databases are authoritative, peer-reviewed, and comprehensive, and include PubMed, PsycINFO, MEDLINE, JSTOR, Scopus, and Web of Science, for interventions contributing to decreasing repeat ED visits for mental health concerns up to November 2022. Two researchers independently screened and reviewed titles, abstracts, and full-text articles that met the inclusion criteria.

Through the use of Covidence software (https://www.covidence.org/ (accessed on 1 February 2022) at Edmonton, AB, Canada), the search strategy was implemented in the electronic databases based on the Preferred Reporting Items for Systematic Reviews and Meta-Analyses (PRISMA) checklist. For each article, two reviewers/authors (WM, RS) were needed to independently screen the titles and abstracts of identified citations, as well as for the full-text review to remove obviously irrelevant material. Disputes between the authors were discussed and resolved by the third author (VA). Data extraction and analysis were performed manually by the authors.

### 2.2. Search Terms

This study followed terms for article extraction after brainstorming and discussion among authors: RCTs (randomized controlled trials), case control, cohort study, case study, case series, analysis and; interventions, strategies, prevention, program, protocol, training, education, suggestions and; emergency visits, readmission, ED, Emergency Department, ER, Emergency Room, acute care, repeat admission and; mental health, psychiatric, disorders, depression, anxiety, panic, bipolar, schizophrenia, psychosis, PTSD and Post-Traumatic Stress Disorder, personality disorder, SUD and Substance Use Disorder, drugs, alcohol, smoking, tobacco.

Boolean operators (AND, OR) were used to combine or exclude keywords in a search. OR is used to combine search terms for the same concept (i.e., emergency department OR emergency room). AND is used to combine different concepts (i.e., mental health AND emergency department AND repeat admission).

### 2.3. Inclusion and Exclusion Criteria

The inclusion and exclusion criteria were based on previous research into available literature and discussions between the authors.

Inclusion criteria:

a. Type of study: ONLY PRIMARY STUDIES. Including RCTs, case-control, cohort study, case study, case series.
b. Interventions: any interventions (clinical and non-clinical) related to the ED (inside the ED, before admission to the ED or after ED discharge) provided to the patients, their families or the medical teams that contribute to decreased ED visits.
c. Outcome: to reduce emergency presentations (repeat) for individuals with mental health instead of the overall trend of the population in general (except if the study is primary study).
d. Mental health conditions/symptoms: all mental health conditions and or symptoms
e. Participants: patients with mental health conditions.
f. Publications are to be in English.

Exclusion criteria:

g. Studies focused on departments other than the emergency room. (e.g., readmission to the psychiatry inpatient).
h. Non-mental health studies (e.g., general health care service).
i. The purpose of the intervention was not to reduce ED admission. This includes improving the ED performance, improving the medical/emergency service, emphasizing the role of the ED, and popularizing understanding about how the ED should be correctly used.

## 3. Results

### 3.1. Search Result

**Figure 1:**
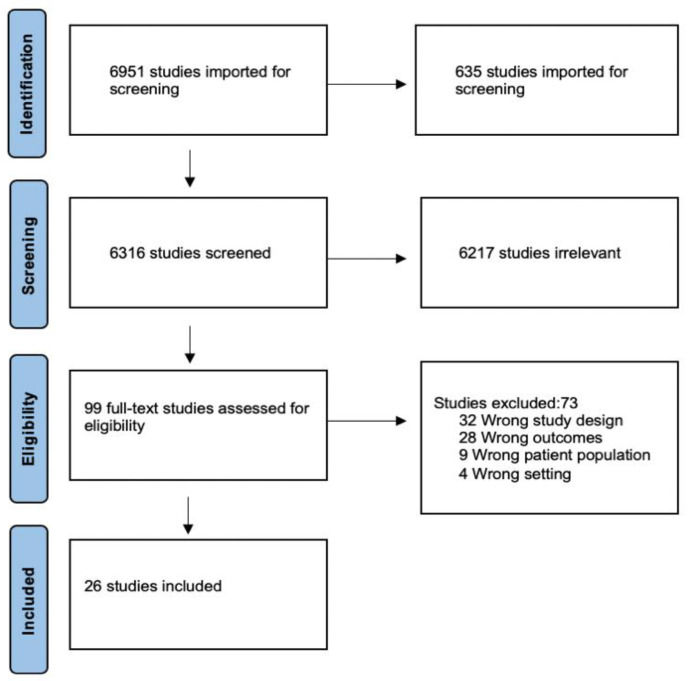
PRISMA flow chart.

The characteristics of the articles are presented in Table 1. A total of 6951 studies was identified from electronic databases searching with Covidence software (Covidence.org: Melbourne, VIC, Australia. Accessed on 1 February 2022). The Covidence software automatically removed 635 duplicate studies from the searched results. The remaining studies (*n* = 6316) were screened against the eligibility criteria identified in the inclusion and exclusion criteria section for this review based on the title and abstract only. Following the title and abstract screening, 99 records were left for full-text screening. As a result of the full-text screening phase, 73 studies were excluded, leaving 26 studies eligible for inclusion in this scoping review. Figure 1 describes the PRISMA—flow diagram summarizing search process and results in detail.

### 3.2. Published Time, Type, and Conducted Countries of the Extracted Studies

**Figure 2:**
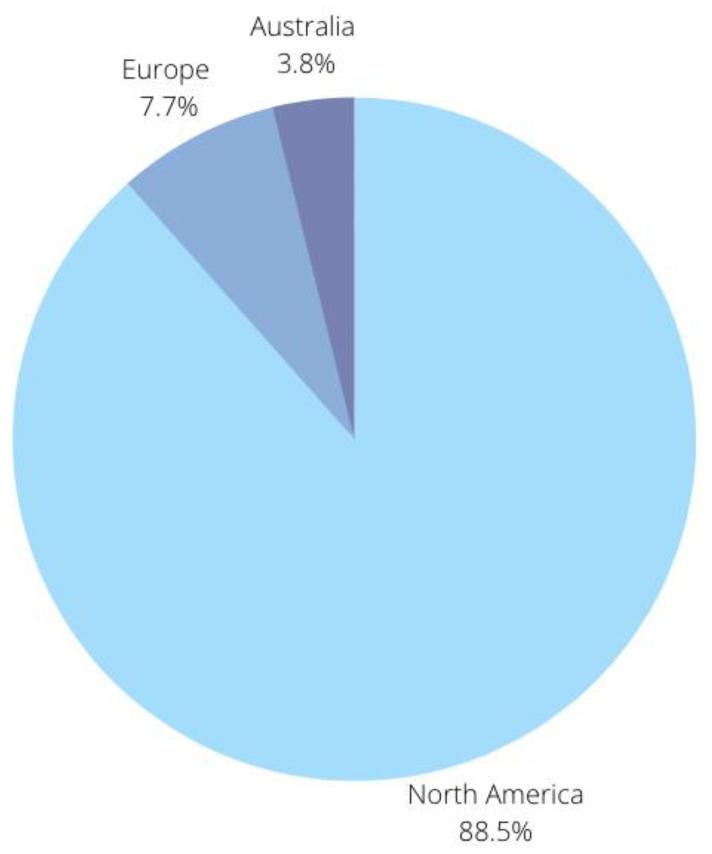
Summary of continents selected for study.

The studies included in this scoping review were published within the last ten years (2010 to 2021). Twenty studies were observational (non-interventional) [25,26,27,28,29,30,31,32,33,34,35,36,37,38,39,40,41,42,43,44] and the rest were interventional studies [45,46,47,48,49,50]. There were twenty-three studies conducted in North America (88.5%) [25,26,28,29,30,31,32,33,34,35,36,39,40,41,42,43,44,45,46,47,48,49], while two were conducted in Europe [27,37], and one in Australia [50] (Figure 2).

**Table 1:** Characteristics of the included studies.

Author and Year	Country	Study Design	Number of Participants	Participants Characteristics (Age/Diagnosis/ Type)	Interventions	Intervention Duration	Key Findings & Conclusion
Abello et al. (2012) [26]	USA	Observational study	48	All patients with psychiatric International Classification Diseases, Ninth Revision, codes (290–312) excluding those with childhood developmental or mental retardation disorders, as those patients present different challenges to adult psychiatric patients	High Alert Program (HAP) is a care plan database created in 2001. The program identifies patients with a history of excessive use of the ED. A 4-level care plan will be created for each individual.	1 year	There was a significant reduction in the number of visits to the ED from the year before program enrollment to the year after enrollment (8.9, before; 5.9, after; *p* < 0.05).
Adaji et al. (2018) [25]	USA	Observational study	5398	All behavioral health patients who presented to the ED between 1 January 2012, and 31 December 2013, and who provided research authorization were included.	Multipayer patient-centered medical home (PCMH) is a patient-centered, team-oriented coordinated care that focuses on the whole patient, including behavioral health needs and conditions.	2 years	PCMH patients (53%) were less likely to be admitted from the ED compared with non-PCMH patients (57%)
Alonso Suarez et al. (2011) [47]	Spain	Observational study	250	All subjects with a diagnosis of schizophrenia that were being treated in 2002 in CCPs in the three CMHS of each participating district.	Continuity-of-Care Programs (CCP) were developed to organize the access to therapeutic resources and treatments available in a territory.	4 years	There was a 40–69% reduction in the proportion of patients visiting the ED, and ED visits. This drop was maintained over the subsequent 3 years of program functioning.
Beere et al. (2019) [50]	Australia	Interventional study	20	Adults (≥18 years) with mental illness and family members or carers of people with mental illness.	Floresco’s integrated service model aims to address the fragmentation of community mental health treatment and support services, which has made it difficult for patients to receive treatment at the appropriate time.	3 years	Decreases in inpatient admissions (20.9% vs. 7.0%), median length of inpatient stay (8 vs. 3 days), ED presentations (34.8% vs. 6.3%) and median duration of ED visits (187 vs. 147 min) were not statistically significant.
Breslau et al. (2018) [44]	USA	Observational study	33,119	Eligible participants have to be aged between 18–64, who were continuously enrolled in Medicaid, and received treatment in a study clinic (either PBHCI or control), during both the baseline and intervention periods.	Primary Behavioral Health Care Integration (PBHCI) program provide screening and monitoring of common chronic physical health conditions along with wellness service, such as smoking cessation or physical activity groups, to their patients.	6 years	ED visits for behavioral health conditions decreased significantly relative to controls in Wave 1 (OR = 0.89), but not in Wave 2.
Celano et al. (2016) [47]	USA	Interventional study	183	Participants had to be at least 18 years; must be fluent in English; with a primary diagnosis of clinical depression, GAD, and/or PD.	Collaborative care (CC) programs is focus on the treatment of depression or anxiety disorder in patients with medical illnesses using nonphysician care managers and consulting team psychiatrists.	6 months	The CC intervention was associated with fewer ED visits but no differences in overall costs.
Chen et al. (2018) [28]	USA	Observational study	920	Older adults ≥ 50 seen as outpatients in an urban medical center serving a low-income community.	Flushing Hospital Medical Center (FHMC) is a low-intensity integrated care model incorporating many elements of successful integrated care programs. It was designed to avoid significantly increasing the burden of responsibility on primary care providers.	2 years	The intervention was associated with reduced costs per visit and reduced likelihood of ED use.
Cummings et al. (2020) [29]	USA	Observational study	40	Participants had to be ≤26 years with ASD diagnosis referred by the ED or by local agencies, including law enforcement.	Access to Psychiatry through Intermediate Care (APIC) aims to address the problems of increasing numbers of visits, lengthening stays, and inadequate specialized intermediate care for people with ASD in our psychiatric ED.	30–650 days	Patients with frequent ED visits spent less time there, because APIC facilitated more rapid discharge to intensive outpatient care, resulting in substantial cost savings.
Das et al. (2021) [49]	USA	Interventional study	1.8 million	The 1.8 million outpatient suicidal ideation and self-harm ED visits in 211 counties, in ten states, from 2006 to 2015.	Continuity of care (CHCs), which is defined by the UDS database as visits per patient as well.	10 years	One unit increase in continuity of mental health care at CHCs corresponds with a 5% decline in ED visits for suicidal ideation/self-harm among whites.
Flowers et al. (2019) [30]	USA	Observational study	58	Patients with 10 or more ED visits in a 6-month period, were 18 years of age or older, and members of the integrated delivery system’s health plan.	Multidisciplinary Care Coordination Program was designed to reduce frequent ED utilization at a single ED. This ED is part of a large, integrated, managed care delivery system in Northern California.	4 years	There was a statistically significant pre-/post difference of 7.7 ED visits. This multidisciplinary care coordination program demonstrated a significant and large reduction in ED visits.
Holder et al. (2017) [31]	USA	Observational study	2661	Children aged 5 to 18 years with a primary diagnosis code for mental illness between 290.0 and 319.0 based on the *International Classification of Diseases*, *Ninth Revision* were included in this study.	Increasing pediatric mental health expertise in the ED.	7 years	After the initiation of the program, ED length of stay decreased significantly from 14.7 to 12.1 h (*p* < 0.001)
Ishikawa et al. (2021) [45]	Canada	Interventional study	N. A	Individual between 0–17 years old whose complaint was under any mental health code in the Canadian ED Information System.	HEARTSMAP is a validated electronic tool that supports ED clinicians in psychosocial assessments and disposition decision making.	3 years	Incremental HEARSTMAP use was associated with a reduction of 1.8 min in ED length of stay and 0.3% in 30-day return visit rate.
Kirby et al. (2021) [32]	USA	Observational study	158	Patients 18–89 years of age who had completed the VA St. Louis Health Care System inpatient rehabilitation program with a diagnosis of OUD between 1 January 2014, and 15 April 2018	Medication-assisted therapy (MAT) for opioid use disorder (OUD) “opioid series”	1 year	Opioid series participation and medication assisted treatment use were independently associated with decreased rates of OUD-related ED visits within 1 year after rehabilitation completion.
Kolbasovsky et al. (2010) [48]	USA	Interventional study	596	Eligible participants had to meet the following primary psychiatric diagnosis (ICD-9 code of 295.00–301.9; 308.3–314.9); aged 18 or older; access to and ability to communicate via telephone; a risk score of 5.0 or higher.	Intensive case management (ICM) services are typically provided by a social worker or nurse responsible for working with the patient, assessing patient needs, ensuring that needs are met, promoting medication and treatment adherence, providing brokerage and advocacy, and linking patients with resources.	1 year	The six-month recidivism rate for baseline group members was 49.67% compared to 22.07% among intervention group members. The program was associated with significantly lower per-member psychiatric ED and inpatient substance abuse costs and utilization.
Kroll et al. (2021) [33]	USA	Observational study	157	Any patient who had previously established medical or surgical care within the hospital system.	Rapid-access ambulatory psychiatric care was developed to provide rapid ambulatory access within a hospital system that cared for a large volume of patients who had demonstrated difficulty in keeping scheduled appointments and had prolonged referral lag times for patients seeking traditional psychiatric care	1 year	For patients who had not previously received ambulatory psychiatric care, ED utilization decreased from 0.68 visits per patient to 0.36.
Lester et al. (2018) [34]	USA	Observational study	4598	All patients who had an ED visit during the specified time intervals and who received a psychiatric consultation during that ED visit were included in the study.	Crisis Assessment Linkage and Management (CALM) model offers crisis intervention care delivered in a designated behavioral health unit located within the medical center but separate from the ED.	3 years	CALM was associated with reductions in median ED and hospital LOS from 9.5 to 7.3 h and 46.2 to 31.4 h, respectively. Mean transformed ED LOS decreased by 32.4% (*p* < 0.001).
Maeng et al. (2020) [35]	USA	Observational study	1213	Patients presenting to ED with behavioral health conditions from three hospitals mentioned in the study.	Psychiatric Assessment Officers (PAO) Model: telepsychiatry is explicitly incorporated as a readily available resource for rural EDs to utilize as deemed necessary.	180 days	The intervention group was associated with an around 36% lower all-cause ED revisit rate during the first 90-day period following the initial PAO treatment. A reduction of similar magnitude (44%) persisted into the subsequent 90 days.
McConville et al. (2018) [36]	USA	Observational study	13.7 million	Nonelderly adults ages 18–64, excluding patients who had any ED visits during the year with Medicare as the expected payer.	Affordable Care Act (ACA) included expanding health coverage; provisions to improve access to health care services by requiring health plans, and by supporting initiatives to improve the coordination of care, particularly for high-need patients.	4 years	After controlling for patient-level characteristics, the odds of being a frequent ED user were significantly lower post ACA for Medicaid-insured patients. Uninsured patients were also less likely to be frequent users post ACA. Privately insured patients had little change.
Nilsson et al. (2014) [37]	Denmark	Observational study	132	Participants had to be aged >18 years; diagnosed with a non-psychotic ICD-10 (18) primary diagnosis (typically depression, anxiety or personality disorders); currently discharged from a psychiatric admission (admitted due to the non-psychotic mental illness)	The intensive transitional post-discharge aftercare (TA) programme was used to fill the gap between concurrent early discharges and specialized outpatient psychotherapeutics with a waiting list of up to two months.	1 year	The number of emergency contacts did not differ significantly between the control group and the study group at any point (rmANOVA; df = 237.1; F = 1612; *p* = 0.2).
Pecoraro et al. (2012) [43]	USA	Observational study	415	Participants had to have clinical suspicion of alcohol and/or drug abuse or dependence; have hospital admission related to alcohol and/or drug abuse; positive result on a drug test AUDIT-PC ≥ 5; primary, secondary, or tertiary diagnosis related to SUD; or self-reported past or current alcohol and/or drug use. Patients above 18 with the ability to provide informed consent forms.	Project Engage, a US pilot program at Wilmington Hospital in Delaware, was conducted to facilitate entry of these patients to SUD treatment after discharge.	3 years	Participants who joined between 1 June 2009–30 November 2009 (*n* = 18): a 38% decrease in ED visits. Who joined between 1 June 2010 and 30 November 2010 (*n* = 25): a 13% decrease in ED visits.
Sullivan et al. (2021) [38]	USA	Observational study	269	Any opioid addiction-related diagnosis in the ED was included in the query followed by chart review to determine if the patient received a referral to the BC.	Buprenorphine Bridge Clinics (BCs) were established in response to the increased need for OUD treatments	8–12 weeks	A 42% reduction in ED visits after patients enrolled. BCs do not reduce ED visits in homeless populations.
Tepper et al. (2017) [46]	USA	Interventional study	1945	Individuals receiving treatment between September 2014–August 2016 for a primary psychotic disorder or bipolar with one or more visits for mental or general medical care before and after the intervention.	Behavioral health home (BHH) provides enhanced access to medical services, care coordination, care transition support, and health promotion.	3 years	BHH patients had fewer total psychiatric total ED visits compared with the control group. Participation in a pilot ambulatory BHH program among patients with psychotic and bipolar disorders was associated with significant reductions in ED visits.
Tillman et al. (2020) [39]	USA	Observational study	157	Patients with psychiatric diagnoses who were hospitalized in medical units other than psychiatry and neuroscience units were excluded.	Pharmacy-driven transitions of care (TOC) services	13 months	Thirty-day psychiatric-associated readmissions, ED presentations, or both occurred in 32.4% and 15.4% of patients in the control and intervention groups. The findings show significant differences in clinical outcomes between patients receiving and not receiving pharmacy-driven transitional interventions.
Uspal et al. (2016) [40]	USA	Observational study	1640	Patients were included in the study if they had a primary discharge diagnosis code consistent with a MH diagnosis (295–302, 308–309, 311–314, v40.2, v40.3, v40.9, v61.0, v61.2, v61.4, v61.8-v61.9, v62.3, v62.4, v62.8, v62.9).	A multistage, multidisciplinary quality improvement (QI) intervention was designed through a multistage, multidisciplinary QI process using Lean methodology	1 years	A significant decrease in mean ED LOS was observed postintervention, from 332 min (95% confidence interval [CI] = 309–353 min) to 244 min (95% CI = 233–254 min.
Wakeman et al. (2019) [41]	USA	Observational study	1353	Adult patients with an SUD diagnosis code, excluding cannabis or tobacco only, receiving primary care at any MGH practice in a 9-month period prior to the site-specific launch of the intervention.	Integrated addiction treatment in primary care	9 months	The mean number of ED visits was lower for the intervention group (36.2 visits vs. 42.9 per 100 patients, *p* = 0.005). Integrated addiction pharmacotherapy and recovery coaching in primary care resulted in fewer ED visits for patients with SUD compared to similarly matched patients receiving care in practices without these services.
Werremeyer et al. (2019) [42]	USA	Observational study	583	All inpatient psychiatric admissions at the city institution between 1 January 2012, and 31 December 2015	Pharmacist-led patient medication education groups (PMEGs)is an intervention in which education is provided to two or more patients about medications or issues related to medication use, with content tailored to the needs of patients in each group	90 days	Attendance at two or more PMEG sessions was associated with a reduction in ED visits for psychiatric reasons (*p* = 0.0433).

### 3.3. Sample Size & Participants’ Characteristics

For the various trials, the sample size varied from *n* = 20 [50] to *n* = 13.7 million [36] and the median sample size was 583. The participants in these studies were all patients with psychiatric diagnoses and who had had emergency visits for reasons related to their mental health issues. Among the 26 papers reviewed, thirteen studies targeted all age groups [25,26,27,33,34,35,38,39,40,42,43,46,49], while nine were conducted on an adult population older than 18 years of age [30,32,36,37,41,44,47,48,50]. Two studies examined the effects of programs designed for children and young adults between the ages of 0–18 years [31,45], and one study targeted patients who were younger than 26 years of age [29]. In one study, participants were above 50 years [28].

### 3.4. Target Conditions and Intervention Type

**Figure 3:**
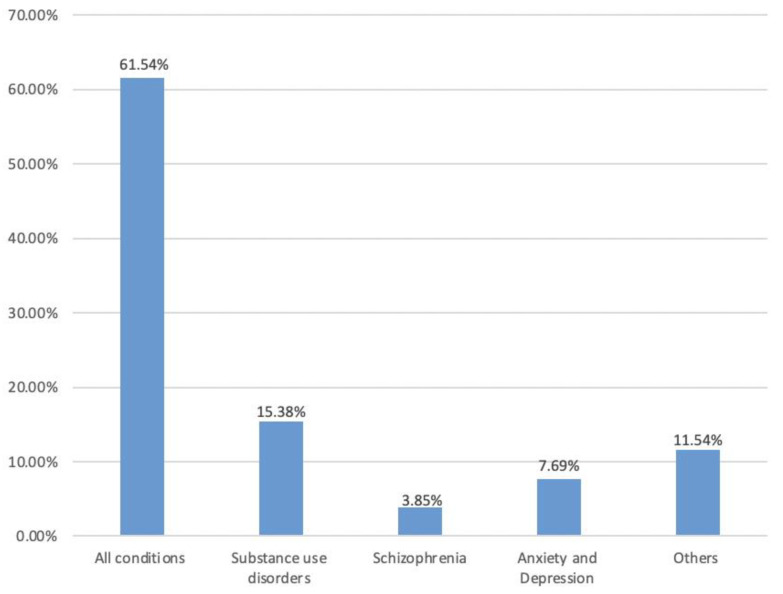
The percentage of studies reporting each psychological condition.

Studies under this review covered a variety of mental health conditions. In total, 16 of the studies addressed interventions targeting all mental health conditions [25,26,28,30,31,33,34,35,36,39,40,42,44,45,48,50]. Other interventions targeting mental health disorders included substance use disorders [32,38,41,43], schizophrenia [27], anxiety and depression [37,47], suicidal ideation and self-harm [49], or other mental health issues [29,46] (Figure 3).

**Figure 4:**
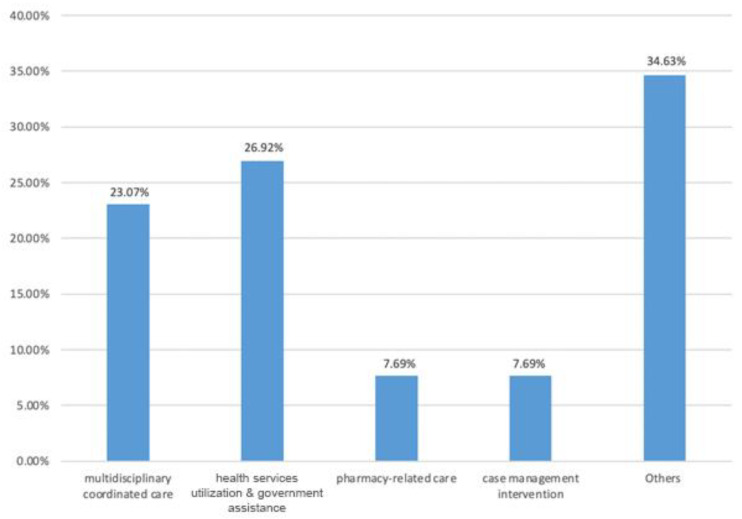
The percentage of studies using each type of intervention.

Among the 26 studies under review, *n* = 6, 23.07% discussed interventions involving multidisciplinary coordinated care, including HAP [26], PCMH [25], PBHCI [44], FHMC [28], MCC [30], and the Floresco’s model [50]; In a total of 7 studies, 26.92% of the studies focused on interventions using various health services resources and seeking government assistance, including HEARTSMAP [45], QI [40], PAO [35], ACA [36], BHH [46], the model of Having Trained Psychiatric Professionals [31], and the rapid-access ambulatory psychiatric care [33]; *n* = 2, 7.69% of the studies addressed pharmacy-related methods, which are TOC [39] and PMEGs [42]; *n* = 2, 7.69.% discussed case management strategies, such as ICM [48] and CALM [34]; the remaining (*n* = 9, 34.63%) emphasized other types of intervention programs (Figure 4).

### 3.5. Outcome and Effectiveness

Of the 26 studies included in this review, the majority (*n* = 25, 96.15%) indicated that the interventions had a positive effect on either self-reported or clinical parameters with regard to reducing ED visits in psychiatry departments. The sustainability of the program effectiveness in reducing ED admissions was reported up to three years following the intervention among CCPs users and one year among MAT therapy for OUD. Cost effectiveness was highlighted in two studies, where CC was effective in reducing ED admissions however no cost savings were achieved, in contrast to the APIC which entailed rapid discharge of the patients to outpatient special care which decreased the service cost.

The studied interventions reduced the length of stay in ED from 1.8 min with HEARTSMAP decision making tool [45], 40 min with Floresco’s integrated service model [50], 88 min with multidisciplinary QI intervention [40], 2.2 h in the CALM model for crises intervention [34], and up to 2.6 h after increasing the mental health expertise in pediatric ED [31].

The number of ED visits after the implementation of the intervention was also reported in some studies. The number of ED visits was reduced from 3 visits after HAP including 4-level care plan intervention [26], 6.7 visits with Integrated addiction treatment in primary healthcare service [41], to 7.7 visits with the Multidisciplinary Care Coordination Program [30]. There was a 5% reduction in ED visits with one unit increase in CHC use, 40–69% reduction with CCP intervention in territorial areas [47], 36% with the PAO model [35], and for OUD there was 13–38% and 42% reduction in ED visits with Project Engage intervention [43] and BCs [38] respectively.

Three studies however, provided limitations to their intervention outcomes [33,42,44]. For example, in Breslau et al.’s study [44], the intervention was conducted in two waves simultaneously, however, only wave one was found to be effective but not wave two. Similarly, Kroll et al. [33] found that their intervention was only effective on patients who had never received it before, and in Werremeyer et al.’s study [42], the authors indicated that attending two or more sessions is necessary for the intervention to be effective. Nilsson’s study, however, was the only study that found no significant differences between intervention and control groups in terms of ED visits after the implementation of the TA program that aimed to close the gap present during the transitioning from ED discharge and the long wait-list outpatient service [37].

## 4. Discussion

### 4.1. Interventions Targeting All the Mental Health Conditions

This review aimed at examining the interventions provided to reduce the admissions to the ED acute care. The review acknowledged 26 research studies that provided interventions to reduce ED admissions among patients with mental health disorders. Through this review, several studies suggested and emphasized the value of primary care supply, multiple providers through service integration, and multidisciplinary coordinated care using different initiatives for reducing the rate of ED visits [25,26,28,30,44,50]. The majority of the interventions were established in North America for all types of mental health diagnoses, with the exception of one study conducted in Australia [50]. From various perspectives, these 16 interventions aimed to reduce patient visits to ED and improve their quality of life.

Service integration may involve a variety of approaches, as discussed by Whiteford et al. [51], for example, formal agreements, joint service planning and provision, single cross-agency care plans, cross-training of staff, shared case records, integrated funding, and co-location of services. Whether being either achieved or not, the process is a continuum [50]. Both the *FHMC Integrated Care Program* [28] and the *Floresco Integrated Service Model* [50] aimed to reduce repeated ED visits with the logic of service integration. The *FHMC* was designed exclusively for old-age low-income individuals who were provided with integrated care, and included stress assessments, psychiatric evaluation, health habits appraisal, and referrals for lifestyle management and mental health illness [28]. However, the *Floresco Integrated Service Model* operated a ‘one-stop’ mental health services hub in partnership with private mental health suppliers and public health service organizations. A comprehensive range of integrated services was provided including mental health services in the community, individualized support, mutual support, self-help groups, family support, and caregiver support [50]. From the literature, the sustainability of healthcare service has long been dependent on service integration, including primary care, since the majority of health and medical services are provided in primary care settings in North America [52]. Therefore, the participants were shown to greatly benefit from these programs and subsequently present less to the ED. 

According to the US Surgeon General’s Report on Mental Health, one-fifth or 21% of children between ages of 9 and 17 years had a diagnosable mental disorder [53]. Mental health issues are one of the leading causes of morbidity and mortality among children and adolescents [53,54,55]. In our review, several interventions were found specifically for children that reduced ED visits significantly. Developed in Canada and the US, *HEARTSMAP* [45] and a multistage, multidisciplinary *QI Intervention* [40] were developed to improve health utilization among young people, through the provision of a holistic health service with dedicated mental health teams who were staffed 24/7 to support youth and young children and evaluate patients and facilitate admissions and discharges [40,45]. Similarly, an effective model of *Having Trained Psychiatric Professionals* [31] has integrated social workers in the Department of Psychiatry to enhance psychiatric expertise in PED. Finding ways to support and fund such services is necessary to address the shortage of mental health services for all patients including young people in the ED. Numerous studies have demonstrated the ED as underequipped and poorly prepared to handle such a growing patient population [31,56,57,58].

Some of the interventions examined the value of allied services such as pharmacy in the reduction of ED visits. The *Pharmacy-driven transitions of care (TOC) services* [39] and the *Pharmacist-led patient medication education groups (PMEGs)* [42] are two examples that have been shown to reduce repeat ED visits, through the development of a pharmacy-related service. Several studies demonstrated that pharmacist involvement in transitions of care improves patient outcomes, supporting the rationale for these interventions [59,60,61]. In addition, governmental support aimed at improving hospital services was demonstrated in this review. For example, the *Rapid-Access Ambulatory Psychiatric Care* [33], the *Affordable Care Act (ACA)* [36], the *Psychiatric Assessment Officers (PAOs)*, and the *Telepsychiatry* plan [35] were designed to reduce repetitive ED visits through the development of walk-in appointments exclusively for psychiatric care, more affordable health insurances, and implementation of technology-based services.

Some other research focused on case management intervention. In healthcare, case management measures, plan, facilitate, and coordinate the management of healthcare-related issues. Communication and resources are utilized in order to meet the health needs of patients and their families [62]. In this review, the *Crisis Assessment Linkage and Management (CALM)* [34] and the *Intensive Case Management (ICM)* [48] were two examples, where evidence suggests that similar services can successfully reduce psychiatric readmissions, as well as repeated and ED visits [49,63,64,65].

### 4.2. Interventions Targeting Substance Use Disorders

Using drugs has long been a topic of discussion in regard to mental health. People who use drugs habitually (PUWD) usually suffer from multiple mental and physical health challenges and have a shorter life expectancy than the general population, which is estimated to be 15 to 20 years [66,67,68]. Additionally, they endure multimorbidity and chronicity of health conditions, which necessitate an understanding of their health care utilization. Compared to a matched cohort, Kendall and colleagues found that PWUD visited the ED seven times more often, which is not surprising since similar findings were demonstrated in numerous other studies [69,70,71,72].

In this review, several studies published interventions that target SUD [32,38,41,43]. In the *Opioid-Specific Education Series* [32] and the *Integrated Addiction Treatment in Primary Care* [41], for example, a variety of treatment methods, including motivational interviewing, counseling, and coaching, cognitive behavioral therapy, and medication education, were applied. Initiatives, such as the *Project Engage* [43] provided at Wilmington Hospital in Delaware facilitate the entry of patients into treatment after they have been discharged. Although the papers included in this review did not provide enough details about this program, it is clear that *Project Engage* is a modified version of BI (Brief Intervention) and SBIRT (Screening, BI, and Referral to Treatment) in which bedside assistance is provided to the clinical team in addressing issues related to SUD [43]. Both studies indicated the significant association of the interventions with decreased rates of SUD-related ED visits [32,43]. Another example is the *Buprenorphine Bridge Clinics (BCs) Program* [38]. A bridge clinic consultation usually takes 1–3 days after a referral from an ED has provided an evaluation and prescribed withdrawal or maintenance treatment by a medical provider where patients in recovery can find support from both medical providers and certified peer advocates or community members with lived experience. A significant reduction in ED visits was observed after the intervention [38]. There is much evidence in the literature that many treatment programs or interventions that aim to treat SUD have been found effective in reducing repetitive ED appointments [73,74,75,76,77,78,79]. The potential of this area is tremendous and research in this area is showing great promise.

### 4.3. Interventions Target Other Mental Health Issues

Based on mental health condition, there was only one intervention designed for patients with schizophrenia, the *Continuity-of-Care Programs (CCP)* [27]; one for autism disorder, the *Access to Psychiatry through Intermediate Care (APIC)* [29]; one for suicidal ideation, the *Continuity of Care (CHCs)* [49]; another for both psychotic and bipolar disorders, the *Behavioral Health Home* (*BHH*) [46]; while two programs targeted anxiety and depression which were the *Collaborative Care (CC) programs* [47] and the *Intensive Transitional Post-Discharge Aftercare (TA) program* [37]. The effectiveness was reported with these programs, albeit not significant for *TA*. The results of other literature studies regarding *TA* indicated a similar result with a reduction in readmissions, but not in ED visits following the intervention [64,80,81,82]. Despite the fact that there is evidence to support the importance of aftercare programs as a way to achieve continuity of care, different studies have revealed controversial results about the outcomes. Research on this topic needs to be conducted in more depth, especially on the effects of aftercare on reducing mental health ED visits.

## 5. Implications for Policy and Practice

For policymakers at all levels, it is important to review and interpret epidemiological data to determine how to improve health care and reduce the number of repetitive ED visits by addressing the root causes. As described in the preceding section, these research studies can assist us in describing patients with mental health issues, understanding their patterns of seeking help, and recognizing perceived barriers to deliver care and opportunities to improve treatment.

Implementing promising and cost-effective health interventions at scale across communities and health systems is essential to achieve population-wide impact. It is pleasing to note that the majority of the interventions that were examined in this scoping review were identified to be scalable and economical. Aside from those interventions addressed in this review, there are other innovations such as mobile text technology which provides evidence-based, cost-effective, geography-independent, and easily scalable methods for delivering psychological interventions to the public with mental health issues. For instance, Text4Mood [83], Text4Hope [84], and Text4Support [22,85] programs, which were implemented in Alberta and Nova Scotia, Canada have proven to provide psychological support services to the general public and successfully reduced psychiatric readmissions and ED visits in various populations. In the end, it is worth emphasizing that government and policymakers must work closely with researchers to develop and implement measures that will reduce the number of repeat visits to the emergency department in the mental health department as well as improve patient quality of life.

## 6. Limitations

It is acknowledged that this scoping review has several limitations. One major limitation relates to the small number of studies that were analyzed and synthesized for qualitative analysis, which raises the importance of garnering the view and feedback of patients along with healthcare providers regarding unmentioned potential effective interventions aimed to reduce ED admission. Although our search covered six databases, the overall search strategy may have been biased toward health and science. It is possible that additional publications could have been found by searching other bibliographic databases. In addition, all studies included in this scoping review were published within the last ten years (2010 to 2021), with the majority of them coming from North America. It is possible that the authors may have missed some studies that were published before this time period in different areas of the world. Additionally, only English-language studies were considered in our search strategy. It is therefore possible that this scoping review left out some valuable studies that were published in other languages. Furthermore, this study focused solely on ED admission and interventions that reduce it among psychiatric patients, therefore other outcomes of the examined studies were not reported here, in order to maintain the aim of the review. Despite the limitations of the study, this scoping review provides an insightful understanding of interventions that may contribute to reducing repeat presentations to hospital ED among patients with mental health issues.

## 7. Conclusions

There has been an increase in patient volumes at hospital ED over the past few decades, resulting in an increased interest in a particular group of patients who attend a disproportionate number of ED visits. After comprehensively examining the published reviews, this scoping review summarized and analyzed the results of 26 published studies that explored interventions to reduce repeat presentations to hospital ED for mental health concerns. These interventions involved comprehensive and multidisciplinary services, that incorporated evidence-based behavioral and pharmacological strategies, emphasizing the case management found to be effective. This review included primarily observational studies and a few interventional studies. Most of the research studies included in this review were conducted in North America between 2010 and 2021.

In addition to those interventions addressed in this review, it is also possible for governments and policymakers to implement an evidence-based, cost-effective, geography-independent, and easily scalable approach for delivering population-level psychological interventions such as Text4Hope [84], Text4Mood [83], and Text4Support [85] which have proven to provide psychological support services to the general public and successfully reduced psychiatric readmissions and ED visits in various populations.

As the majority of studies at the moment focus on examining interventions in relation to hospital readmission, considering the tremendous burden caused by recurrent emergency room visits on the health system and patients, it could be interesting and meaningful for future studies to investigate more the risk and protective factors that influence the frequency and intensity of ED visits for mental health concerns, in order to provide a better diagnostic and prevention tool.

## Data Availability

Not applicable.
